# Direct T Cell Activation via CD40 Ligand Generates High Avidity CD8^+^ T Cells Capable of Breaking Immunological Tolerance for the Control of Tumors

**DOI:** 10.1371/journal.pone.0093162

**Published:** 2014-03-24

**Authors:** Ruey-Shyang Soong, Liwen Song, Janson Trieu, Sung Yong Lee, Liangmei He, Ya-Chea Tsai, T.-C. Wu, Chien-Fu Hung

**Affiliations:** 1 Department of Pathology, Johns Hopkins Medical Institutions, Baltimore, Maryland, United States of America; 2 Department of Obstetrics and Gynecology, Johns Hopkins Medical Institutions, Baltimore, Maryland, United States of America; 3 Department of Molecular Microbiology and Immunology, Johns Hopkins Medical Institutions, Baltimore, Maryland, United States of America; 4 Department of Oncology, Johns Hopkins Medical Institutions, Baltimore, Maryland, United States of America; 5 Department of General Surgery, Chang Gung Memorial Hospital at Keelung, Keelung City, Taiwan; 6 Chang Gung University, College of Medicine, Taoyuan, Taiwan; 7 Pharmacy School of Fudan University, Shanghai, China; 8 Department of Pharmacology and Toxicology, Shanghai Institute of Planned Parenthood Research, Shanghai, China; 9 Department of Obstetrics and Gynecology, Shanghai Tenth People's Hospital of Tongji University, Shanghai, China; 10 Johns Hopkins University, Baltimore, Maryland, United States of America; 11 Department of Internal Medicine, Korea University Medical Center, Seoul, South Korea; Federal University of São Paulo, Brazil

## Abstract

CD40 and CD40 ligand (CD40L) are costimulatory molecules that play a pivotal role in the proinflammatory immune response. Primarily expressed by activated CD4^+^ T cells, CD40L binds to CD40 on antigen presenting cells (APCs), thereby inducing APC activation. APCs, in turn, prime cytotoxic T lymphocytes (CTLs). Here, two tumor-associated antigen (TAA) animal models, p53-based and GP100-based, were utilized to examine the ability of CD40-CD40L to improve antigen-specific CTL-mediated antitumor immune responses. Although p53 and GP100 are self-antigens that generate low affinity antigen-specific CD8^+^ T cells, studies have shown that their functional avidity can be improved with CD40L-expressing APCs. Therefore, in the current study, we immunized mice with a DNA construct encoding a TAA in conjunction with another construct encoding CD40L via intramuscular injection followed by electroporation. We observed a significant increase in the antigen-specific CTL-mediated immune responses as well as the potent antitumor effects in both models. Antibody depletion experiments demonstrated that CD8^+^ T cells play a crucial role in eliciting antitumor effects in vaccinated mice. Furthermore, we showed that *in vitro* stimulation with irradiated tumor cells expressing both TAA and CD40L improved the functional avidity of antigen-specific CD8^+^ T cells. Thus, our data show that vaccination with TAA/CD40L DNA can induce potent antitumor effects against TAA-expressing tumors through the generation of better functioning antigen-specific CD8^+^ T cells. Our study serves as an important foundation for future clinical translation.

## Introduction

CD40 and CD40 ligand (CD40L) are costimulatory molecules typically expressed on antigen presenting cells (APCs) and T cells, respectively. CD40 is a 48 kDa transmembrane glycoprotein cell surface receptor that binds to the 34–39 kDa type II integral membrane protein CD40L. The interaction between these tumor necrosis factor α (TNF-α) receptor family members is important for T cell activation. This contact stimulates high levels of IL-12 production by dendritic cells (DCs), promoting a Th1 immune response [1]. It also triggers prolonged MHC-antigen complex presentation, inflammatory cytokines, and DC survival. In the classic model, DCs are licensed by CD4^+^ T helper cells, via CD40-CD40L interaction and then activate cytotoxic T lymphocytes (CTLs) (for review see [2]). However, an alternate mechanism via CD40-CD40L may activate CTLs. More recently, it has been shown that CD40L expression on activated DCs, which is inducible by viruses, can directly prime CTLs [3], [4]. These CTL activation mechanisms represent opportunities to enhance antigen-specific CD8^+^ T cell-mediated immune responses.

Indeed, a variety of tumor therapies have been developed, which exploit the CD40-CD40L costimulatory effects (for review, see [5]). For example, an agonistic anti-CD40 antibody may serve as an ideal therapy to supplement standard cancer treatments such as chemotherapy (for review, see [6]). Additionally, various humanized anti-CD40 antibodies have been tested and are being tested in clinical trials, many in combination with chemotherapy [7], [8]. Furthermore, adenoviral cancer vaccines encoding TAAs targeted to CD40 on DCs are currently in development [9], [10] (for review, see [11]). While treatments manipulating CD40-CD40L are promising, it remains important to target cancer treatments specifically to tumors.

Cancer immunotherapy via tumor-specific T cell stimulation has several advantages. First, T cells can target antigens specifically expressed by tumors but not in normal cells and can thereby avoid non-specific damage to normal tissues. Second, effector T cells can proliferate efficiently after coming in contact with tumors or APCs, leading to further activation. After clearing the pathogen, T cells differentiate into memory T cells, which are responsible for long-lasting immunity [1], [12]–[14]. Tumor antigens have been identified in nearly every human cancer. Among those antigens is tumor suppressor protein p53, a highly conserved genome-guarding mechanism. p53 serves to halt the cell cycle during normal instances of DNA damage allowing time for repair [15]–[17]. However, for this very reason, p53 is frequently inactivated via sequence mutations in more than 50% of common human cancers. The mutations in p53 result in significant upregulation of p53 in tumor cells. Consequently, p53 represents a promising target for cancer immunotherapy [12]–[14], [18], [19]. Similarly upregulated in a subset of melanomas is GP100 [17], [20]. This protein is well studied and has already been used to develop a therapeutic peptide vaccine composed of amino acids 209–217. Therefore, these antigenic systems are potentially suitable models for further examining the role CD40L in T cell activation. Like GP100, p53 is an endogenous antigen that is poorly immunogenic. Therefore, attempts at immunization against these endogenous tumor antigens generate, at most, low affinity antigen-specific CD8^+^ T cells. This issue continues to be a bottleneck for tumor immunotherapy.

In the current study, we developed a naked DNA vaccine delivered via intramuscular (IM) injection followed by electroporation. Electroporation is a common DNA vaccine administration technique utilized in our previous studies due to its ability to improve transfection efficiency to generate potent antigen-specific CD8^+^ T cell-mediated immune responses [18], [19], [21], [22]. Using this system, we found that vaccination with a DNA vaccine encoding p53 or GP100 in conjunction with DNA encoding CD40L generated high avidity antigen-specific CD8^+^ T cells in vaccinated mice compared to vaccination with the p53 or GP100 DNA alone. Functional avidity is the relative frequency of IFN-γ^+^ CD8^+^ T cells induced by titrated peptide concentrations [23]–[25]. Furthermore, co-administration of DNA encoding CD40L with these antigen specific DNA vaccines was able to break immune tolerance resulting in better tumor control and protection. We also found that *in vitro* stimulation of T cells with CD40L–expressing irradiated tumor cells loaded with antigenic peptide significantly improved the generation of tumor-specific cytotoxic CD8^+^ T cells responsible for tumor clearance. We determined that repeatedly simulating T cells with tumor cells co-expressing CD40L could change T cell functional avidity as well as increase anti-apoptotic and decrease pro-apoptotic signaling. In addition, T cells displayed an increased T cell receptor (TCR) density. The implication of our study for future clinical translation will be discussed.

## Materials and Methods

### Ethics Statement

All animal procedures used in this study were performed according to protocols approved for this specific study and in accordance with recommendations for the proper use and care of laboratory animals by Johns Hopkins University Animal Care and Use Committee (approved protocol number MO11M509). All cell lines were established and maintained with approved protocols. Mice were sacrificed for the purpose of this study using CO_2_ in accordance with the animal protocol. Regarding human endpoint standards, mice showing severe distress or bearing tumors that exceeded 1.5 cm in diameter were euthanized with CO_2_ in accordance with the animal protocol.

### Mice

6- to 8-week-old C57BL/6 female mice were purchased from the National Cancer Institute-Frederick Animal Production Area (Frederick, MD). Pmel-1 TCR transgenic mice [17], [20] under thy1.1 background were purchased from The Jackson Laboratory. Animals were kept in the oncology animal facility of the Johns Hopkins Hospital (Baltimore, MD).

### Cell lines

MC38 is a murine colon adenocarcinoma cell line that highly expresses mouse p53 protein (mp53) and was established and provided by Dr. Steven Rosenberg with approval to create and maintain the cell line (National Cancer Institute, NIH, Bethesda, MD) [21], [22], [26]. TC-1 cells were produced in our laboratory and maintained under approved protocols as previously described [27], [28]. MC38 cell lines were cultured *in vitro* in DMEM supplemented with 10% fetal bovine serum, 50 IU/mL of penicillin/streptomycin, 2 mM L- glutamine, 1 mM sodium pyruvate, and 2 mM non-essential amino acids. TC-1 cell lines were cultured *in vitro* in RPMI 1640 supplemented with 10% fetal bovine serum, 50 IU/mL of penicillin/streptomycin, 2 mM L- glutamine, 1 mM sodium pyruvate, and 2 mM non-essential amino acids. All cell lines were grown at 37°C with 5% CO_2_. Luciferase- and GFP-expressing MC38 (MC38-GFP/Luc) cells were generated by transduction with a lentivirus containing luciferase and GFP [29]. Lentiviral vector pCDH1-luc-EF1-GFP [29] was transfected into the Phoenix packaging cell line using lipofectamine (Invitrogen, Carlsbad, CA, USA) and the virion-containing supernatant was collected 48 hrs after transfection. The supernatant was then filtered through a 0.45 mm cellulose acetate syringe filter (Nalgene, Rochester, NY, USA) and used to infect MC38 cells in the presence of 8 mg/mL Polybrene (Sigma-Aldrich, St Louis, MO, USA).

The CD40L-and GFP-expressing TC-1 cell line (TC-1/CD40L) was also generated by the same method as the lentiviral vector pCDH1-CD40L-GFP [30] (**Figure S1**). The luciferase-expressing B16 cell line (B16-Luc) [31] was established and provided by Dr. Sam T. Hwang (NIH) with approval to create and maintain the cell line. Transduced cells were isolated using preparative flow cytometry gated on the GFP signal. Irradiated splenocytes or irradiated TC-1 cells (iTC-1) were used as feeder cells in the study. The iTC-1 cells were loaded either with mp53-specific CTL epitope (aa 232–240, KYMCNSSCM) (mp53-iTC-1) 1 μg/ml or mGP100_25–33_ EGSRNQDWL (mGP100-iTC-1)1 μg/ml, the T cell epitope under H-2K^b^ background as previously mentioned [20], [32]. The same method was applied to irradiated TC-1/CD40Lcells (iTC-1/CD40L) to generate mp53-iTC-1/CD40L and mGP100-iTC-1/CD40L respectively.

### DNA constructs

PcDNA3-mp53 and pCDH1-luc-EF1-GFP were described previously [32]. pcDNA3-CD40L [33] was kindly provided by Dr. Thomas J. Kipps at the University of California, San Diego. PCDH1-CD40L-GFP was generated by removing CD40L from pcDNA3-CD40L and cloning it into the PCDH1-MCS1-EF1-copGFP vector (System Biosciences) at XboI/EcoRI cloning site. For the generation of pcDNA3-mGP100, DNA fragments encoding the full length of mouse GP100 were generated by RT-PCR using mouse B16 melanoma RNA and a pair of primers, AAATCTAGAGCCACCATGGTGGGTGTCCAGAGAAGG and TTTGAATTCTTAACCATCCGGTAGTACCTTTTG. The amplified DNA was further cloned into the XbaI and EcoRI sites of pcDNA3.

### Luciferase-based bioluminescence imaging

Firefly luciferase (Luc) and the substrate coelenterazine (Sigma) were used to test for luciferase activity *in vitro* [26], [30]. For *in vitro* cell staining, tumor cells expressing luciferase were added to 24-well plates and incubated for 12 hours. Subsequently, the antigen-specific T cell line was added in titrated ratio to each well and left overnight at 37°C. The wells were washed twice with PBS before coelenterazine was added. Bioluminescence of the cells was detected using the IVIS Imaging System 200 Series. The region of interest from displayed images was designated and quantified as total photon counts using Living Image 2.50 software (Xenogen).

### In vitro activation of E7-, mp53- and Pmel-1-specific CD8^+^ T cell lines

We used previously described methods to generate E7-specific CD8^+^ T cell lines [28], [34]. To generate the mp53-specific CD8^+^ T cell line, splenocytes were isolated 7 days after the last vaccination from C57BL/6 mice vaccinated intramuscularly with the 10 μg/mouse of pcDNA3-mp53 followed by electroporation 3 times at 1 week intervals and cultured in culture media under IL-2 (20 IU/mL) in the presence imTC-1 or imTC-1/CD40L. In order to increase the population of mp53-specific CD8^+^ T cells, the stimulated T cells were stained with CD8/H-2K^b^ KYMCNSSCM [35] pentamer (Immunex Co.) and sorted for further culture. We successfully generated a higher percentage of mp53-specific CD8^+^ T cells (**Figure S2**). For Pmel-1-specific CD8^+^ T cells, splenocytes from Pmel-1 TCR-transgenic mice were depleted of erythrocytes by hypotonic lysis and cultured in culture media with IL-2 (2 IU/mL) in the presence of iTC-1 or iTC1/CD40L loaded with 1 μg/ml hGP100_25–33_ KVPRNQDWL peptide [20]. The T cells were re-stimulated every 5–7 days.

### Electroporation-mediated DNA vaccination

The square-wave electroporator (Model 830; BTX) consists of an electrode array, a pulse generator, and a foot pedal for pulse activation. Disposable 30G needles (Becton-Dickinson, Franklin Lakes, NJ) were used in the center of the electrode grid to administer DNA plasmid equidistant between the two needle array electrodes (BTX, San Diego, CA). Mice were injected in the tibialis muscle of the shaved hind leg with activation of the pulse generator as instructed by the manufacturer and as previously described [32], [34]. 10 μg of DNA plasmid was diluted in a total volume of 20 μL of PBS for each injection. When vaccination schedules required a booster vaccination, the contralateral leg was used for vaccination. Subsequent vaccinations used alternating hind legs.

### Phenotype of T cells and intracellular staining

To analyze the phenotype of T cells, cell suspensions were stained with the following antibodies: anti-CD8 PE/APC, FITC (53–6.7), anti-Fas FITC, anti-FasL PE (MFL-3), anti-thy1.1 PE, anti-active caspase 3 PE, and anti-Annexin V PE from BD, anti-CD40 (HM40-3), anti-CD40L, anti-νβ13 TCR FITC (MR12-3) from eBioscience. The H-2D^b^ E7 tetramer (gift from NIH), H-2K^b^ KYMCNSSCM pentamer, and H-2K^b^ SIYRYYGL pentamer (Immunex Co.) were used for cell surface analysis. Cell surface marker staining for CD8, intracellular cytokine staining for IFN-γ and FACScan analysis were all performed under the same conditions as previously mentioned [32], [35]. Three mice in each group were vaccinated with 10 μg of pCDNA3 vector, pCDNA3-mp53, pCDNA3-CD40L, or pCDNA3-mp53/pCDNA3-CD40L combined vaccine by electroporation and received two booster vaccinations every 7 days. One week after the last vaccination, splenocytes (2×10^6^) were collected and incubated with p53 peptide 232–40 (1 μg/mL) in 24 well plates under interleukin-2 stimulation for one week as previously mentioned [35], [36]. Then, the numbers of IFN-γ-secreting CD8^+^ T cells were analyzed by FACScan cytometry after adding Golgi plug (1 μg/mL, BD Pharmingen) for 8 hours. Analysis was performed on a Becton-Dickinson FACScan with CELLQuest software (Becton-Dickinson Immunocytometry System, Mountain View, CA) and Flowjo 5.6 software.

### In vivo tumor protection

For all tumor experiments, the MC38 murine colon adenocarcinoma cell line was used. Five mice per group were vaccinated with 10 μg/mouse of pCDNA3 vector, pCDNA3-mp53, pCDNA3-CD40L, or 10 μg/mouse of pCDNA3-mp53 and 10 μg/mouse of pCDNA3-CD40L combined DNA vaccine via electroporation followed by three weekly booster vaccinations using the same method. One week after the last vaccination, each mouse was challenged with 2×10^5^ MC38 cells injected subcutaneously. All mice were observed for 80 days to monitor tumor progression. Tumor growth was measured by electronic calipers twice a week and the percentages of surviving mice were recorded. Tumor volume was approximated by the following formula: length x width^2^ x 0.5. Animals showing severe distress or bearing tumors that exceeded 1.5 cm in diameter were euthanized in accordance with the animal care protocol.

### In vivo Ab-depletion experiment

Five mice in each group were vaccinated with combined pCDNA3-mp53/pCDNA3-CD40L DNA vaccine as previously mentioned. After the last vaccination, CD4, CD8, or NK1.1 cell depletion (100 μg/mouse) was initiated with antibody treatments at 2 day intervals one week before tumor challenge. After tumor challenge, antibody was given every 7 days, until day 42. Tumor growth was determined by electronic caliper twice per week and the percentages of surviving mice were recorded. The mAb GK1.5 was used for CD4 depletion, mAb 2.43 was used for CD8 depletion, and mAb PK136 was used for NK1.1 depletion as previously mentioned [37].

### In vivo tumor treatment

Five mice per group were challenged subcutaneously with 2×10^5^ MC38 cells/mouse. Five days after tumor challenge, mice were vaccinated with 10 μg/mouse of pCDNA3 vector, 10 μg/mouse pCDNA3-mP53, or 10 μg/mouse of pCDNA3-mp53 and 10 μg/mouse of pCDNA3-CD40L combined DNA vaccine via electroporation. Mice received a booster vaccination at 5 day intervals for a total of three vaccinations in alternating hind legs. Tumor size was measured with electronic calipers and tumor volume was calculated starting on day 5. Measurements were recorded twice per week.

### Generation of mGP100 T cell chimeric mice

To generate mGP100 T cell chimeric mice, C57BL/6 mice with Thy1.2 background were subjected to bone marrow depletion by irradiation with a sub-lethal dose of 600 cGy [38]. 5×10^6^ splenocytes from Pmel-1 TCR transgenic mice (Thy1.1 background) were adoptively transferred into C57BL/6 mice immediately after irradiation.

### Western Blot of Bcl-XL protein expression in T cells

T cells were collected from *in vitro* culture. After centrifugation, the T cell pellet was lysed with mammalian protein extraction reagent (Thermo Scientific, IL). 50 μg of protein from the T cell lysate was resolved by SDS-PAGE and transferred to nitrocellulose membranes (GE Bioscience). After blocking with 5% skim milk in PBS–0.1% Tween 20 (PBST) for 1 hour at room temperature, membranes were incubated with anti-Bcl-XL rabbit polyclonal antibodies 1∶500 (Santa Cruz, CA) at 4°C overnight. Membranes were then washed with PBST and incubated with anti-rabbit horseradish peroxidase (HRP)–conjugated secondary antibody before visualization with ECL plus (GE Bioscience). Anti-β-actin (Sigma; 1∶5,000) antibodies were used as a loading control. All antibodies were diluted in blocking buffer.

### Quantitative real-time PCR

mRNA from T cells was extracted using Trizol as previously described [30]. First strand cDNA was synthesized by RSIII, according to the manufacturer's protocol (Invitrogen). Then, the first strand cDNA was used for quantitative real-time PCR using IQ SYBR Green (Invitrogen) on the MyiQ real-time detection system (Bio-Rad) following the manufacturer's protocol. The forward and reverse primers for Bcl-XL, Bcl-2, Bim, β-Actin and TCR Vβ13 have been previously described [39], [40]. Experimental data was normalized to the control group using CFX manager software (Bio-Rad) following the manufacturer's protocol.

### Statistical analysis

All data are expressed as mean ± S.E. where indicated. Comparisons between individual data points for intracellular cytokine staining with flow cytometric analysis and tumor treatment were made using a Student's t-test. In the tumor protection experiments, the principal outcome of interest was duration until development of a tumor ≥1.5 cm. The event time distributions for different mice were compared using the Kaplan–Meier method and the log-rank statistic by SPSS 17 software. All p-values <0.05 were considered significant.

## Results

### Co-administration with CD40L DNA with mp53 DNA significantly increases mp53-specific CD8^+^ T cells immune responses in vaccinated mice

**Figure 1:**
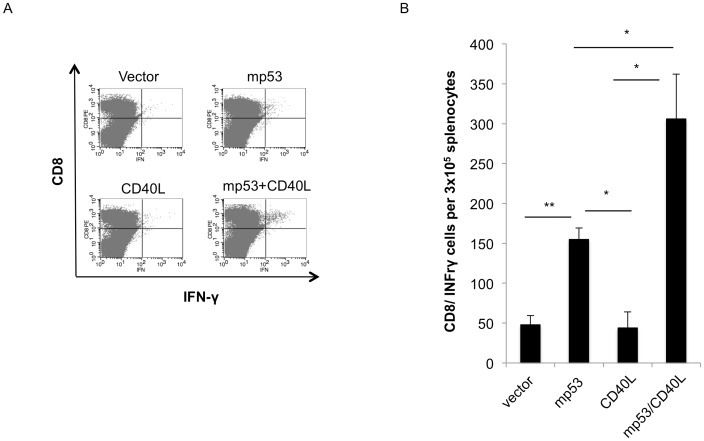
Characterization of mp53-specific CD8^+^ T cell response following vaccination with mp53 DNA with or without coadministration of CD40L DNA. C57BL/6 mice received intramuscular vaccination followed by electroporation with mp53 DNA plasmid in conjunction with or without CD40L DNA three times at one week interval. Empty DNA or CD40L DNA was included as a control. One week after the last vaccination, splenocytes from vaccinated mice were isolated and cultured with p53 epitope 232 with IL-2 stimulation for one week. The presence of mp53-specific CD8^+^ T cells was determined using CD8 and intracellular IFN-γ staining followed by flow cytometry analysis. (A) Representative flow cytometry analysis. (B) Bar graph depicting the number of IFN-γ/CD8+ cells per 3×10^5^ splenocytes. Data presented as mean ± S.E. (*p<0.05; **p<0.01).

In order to test whether co-administration of CD40L DNA could lead to activation of antigen-specific CD8^+^ T cells against endogenous antigen, C57BL/6 mice were vaccinated with mp53 DNA or mp53 and CD40L DNA plasmids. Briefly, the DNA plasmid was administered by intramuscular (IM) injection followed by electroporation three times at one-week intervals. Seven days after the last vaccination, splenocytes from each group were obtained and stimulated with mp53-specific CTL epitope (aa 232–240, KYMCNSSCM) (1 μg/mL) and IL-2 (2 IU/mL) for one week, followed by intracellular cytokine staining. As shown in **Fig. 1A and B**, mice vaccinated with pcDNA3-mp53/pcDNA3-CD40L DNA plasmid vaccines generated significantly higher numbers of mp53-specific CD8^+^ T cells compared to mice vaccinated with only pcDNA3-mp53 DNA or pcDNA3-CD40L. Our data indicate that the number of mp53-specific CD8^+^ T cells generated by mp53 DNA vaccine is greatly enhanced by co-administration with CD40L DNA plasmid.

In order to determine whether CD40L costimulation is effective against a foreign tumor antigen, human papillomavirus type 16 (HPV-16) E7 protein, groups of C57BL/6 mice were vaccinated with pcDNA3-CRT-E7 DNA or pcDNA3-CRT-E7/pcDNA3-CD40L DNA plasmid via IM injection followed by electroporation two times at a one week interval. One week after the last vaccination, peripheral blood was drawn from the tail vein for CD8 and MHC class 1 E7 tetramer staining. Splenocytes were also isolated for intracellular staining. No significant difference was observed between T cells obtained from pcDNA3-CRT-E7 or pcDNA3-CRT-E7/pcDNA3-CD40L vaccinated mice (**Fig. S3 A–D**). We further examined E7-specific T cell activation *in vitro*, following interaction with TC-1 or TC-1/CD40L. There was no significant difference in the numbers of IFN-γ^+^ CD8^+^ T cells between both groups. (**Fig. S3 E–F**). Taken together, our data suggest that co-administration of DNA encoding CD40L with antigen-specific DNA vaccine were able to enhance antigen-specific CD8^+^ T cell against endogenous tumor antigen but not foreign tumor antigen.

**Figure 2:**
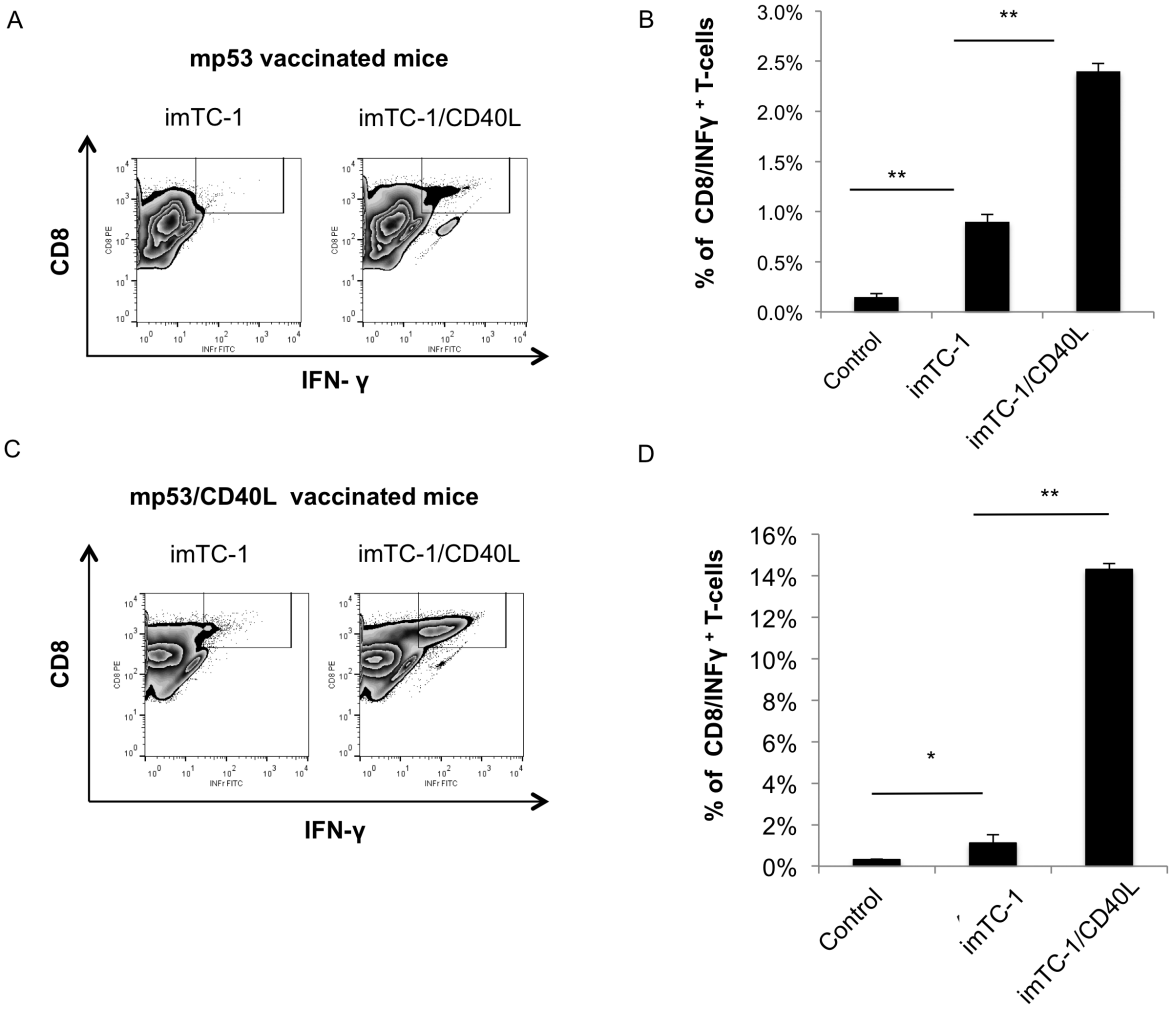
Characterization of mp53-specific CD8^+^ T cell expression following in vitro stimulation with irradiated tumor cells with or without CD40L expression. Splenocytes from mice vaccinated with mp53 DNA and incubated with mp53-iTC-1 or mp53-iTC-1/CD40L for 7 days with IL-2. The presence of mp53 specific CD8^+^ T cells were characterized by staining for CD8 and IFN-γ and analyzed by flow cytometry. (A) Representative flow cytometry analysis (B) Bar graph depicting the percentage of mp53-specific CD8^+^ T cells. Splenocytes from mice vaccinated with mp53 and mp53/CD40L DNA plasmids were isolated and incubated with mp53-iTC-1 or mp53-iTC-1/CD40L for 7 days with IL-2. The number of mp53-specific CD8^+^ T cells was then characterized by intracellular cytokine staining for IFN-γ and CD8 followed by flow cytometry analysis. (C) Representative flow cytometry analysis. (D) Bar graph depicting the percentage of mp53-specific CD8^+^ T cells among all T cells. Data presented as mean ± S.E. (*p<0.05; **p<0.01).

Next, we stimulated T cells isolated from mice vaccinated with mp53 or mp53/CD40L DNA to assess their response *in vitro*. Splenocytes from mp53 DNA vaccinated mice were stimulated with mp53-iTC-1 or mp53-iTC-1/CD40L for 3 cycles and then assessed by intracellular cytokine staining. As shown in **Fig. 2A and B**, the splenocytes stimulated with mp53-iTC-1/CD40L had a significantly higher number of mp53-specific CD8^+^ T cells compared to splenocytes stimulated with mp53-iTC-1 (*p*<0.01). When mp53-specific CD8^+^ T cells from mice vaccinated with mp53/CD40L DNA were stimulated under the same conditions, the mp53-specific CD8^+^ T cells incubated with mp53-iTC-1/CD40L had significantly more IFN-γ^+^ production compared to those incubated with mp53-iTC-1 tumor cells (*p*<0.01) (**Fig. 2C and D**). Taken together, these data indicate that mp53-specific CD8^+^ T cells from mice vaccinated with mp53/CD40L DNA could generate a striking immune response *in vivo* and *in vitro*.

### Mice vaccinated with combination mp53/CD40L DNA vaccines display superior protection against murine mp53-expressing MC38 tumors compared to mice vaccinated with mp53 DNA vaccine alone

**Figure 3:**
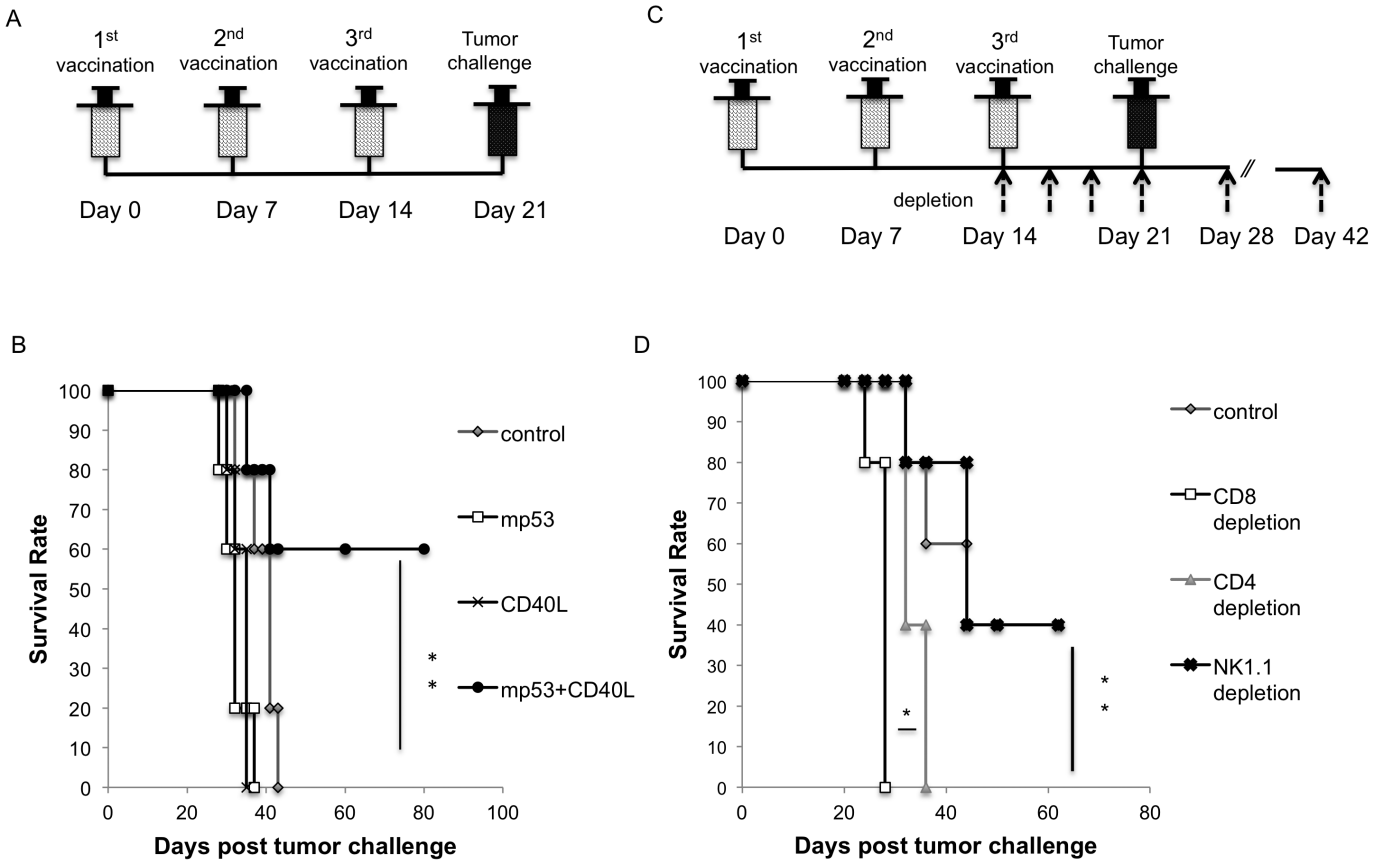
In vivo tumor protection experiments in mice vaccinated with mp53 or mp53/CD40L DNA by electroporation. Mice (n = 5) were immunized with various DNA vaccines (mp53, CD40L, or mp53/CD40L) three times at one week intervals and then challenged with MC38 tumor cells (2×10^5^/mouse). 1 week later, mice were monitored for survival following tumor challenge. (**A**) Schematic diagram depicts the vaccination and tumor challenge schedule. (**B**) Kaplan-Meier survival analysis. Data presented as mean ± S.E. (**p<0.01). (**C**) Mice (n = 5) were immunized with mp53/CD40L DNA vaccine via intramuscular injection with electroporation using the same regimens and challenged with 2×10^5^ MC38 cells per mouse. Anti-CD4, anti-CD8, anti-NK1.1 antibodies (100 μg/mouse) were administered every other day, beginning one week before tumor challenge. Following tumor challenge, antibodies were administered every 7 days and the treatment was terminated 30 days after tumor challenge. Schematic diagram depicts the vaccination and depletion schedule. (**D**) Kaplan-Meier survival analysis. Data presented as mean ± S.E. (*p<0.05; **p<0.01).

Based on the immunology findings, we examined whether the vaccination strategy combining pcDNA3-mp53 and pcDNA3-CD40L DNA plasmids could control tumors highly expressing mp53 *in vivo*. Briefly, C57BL/6 mice (five in each group) were vaccinated intramuscularly with 10 μg/mouse of pCDNA3 vector, pcDNA3-mp53, pcDNA3-CD40L, or pcDNA3-mp53/pcDNA3-CD40L (10 μg each) combined vaccine via electroporation. Mice received booster vaccinations on days 7 and 14. On day 21, mice were inoculated subcutaneously with 2×10^5^ MC38 tumor cells and were closely observed for palpable tumor development. The treatment schedule is outlined in **Fig. 3A**. Our results showed that mice vaccinated with the pcDNA3-mp53/pcDNA3-CD40L combined vaccine had small tumor volume (**Fig. S4A**) and a significantly higher survival rate (*p*<0.01) in comparison to other treatment groups (**Fig. 3B**). After 80 days, 60% of the mice receiving the combined pcDNA3-mp53/pcDNA3-CD40L vaccination were tumor free.

We next determined which lymphocytic group was responsible for the tumor protection effect elicited by the mp53/CD40L combined DNA vaccination. Mice (five per group) were vaccinated three times with pcDNA3-mp53/pcDNA3-CD40L DNA vaccine via electroporation, according to the schedule in **Fig. 3C**. Following the last vaccination, anti-CD4, anti-CD8 or anti-NK1.1 mAb was injected intraperitoneally, every other day. On day 28, MC38 tumor cells (2×10^5^) were inoculated subcutaneously and the tumor volume was measured twice per week. Mice depleted of CD8^+^ cells had larger tumor volume (**Fig. S4B**) and significantly poorer survival compared to mice depleted of CD4^+^ or NK1.1^+^ cells (**Fig. 3D**). These results suggest that CD8^+^ T cells are essential for the antitumor effects generated by the mp53/CD40L vaccine.

### pCDNA3-mp53 and pCDNA3-CD40L combined DNA vaccine DNA vaccine induces a positive therapeutic effect in MC38 tumor bearing mice

Using the therapeutic model, we compared the different DNA vaccines against the MC38 tumor cell line. As outlined in **Fig, S5A**, mice were injected with 2×10^5^ MC38 tumor cells subcutaneously on day 0. Mice were subsequently vaccinated three times with either vector only, pCDNA3-mP53, or pcDNA3-mp53/pcDNA3-CD40L combined vaccine by IM administration followed by electroporation. Tumor volume was measured twice per week. As shown in **Fig. S5B**, mice vaccinated with the pcDNA3-mp53/pcDNA3-CD40L combined vaccine elicited sustained antitumor responses compared with the pCDNA3mP53 and vector only vaccinated groups.

### CD8^+^ T cells from mice vaccinated with mp53/CD40L DNA demonstrated potent mp53-specific cytotoxic effects

**Figure 4:**
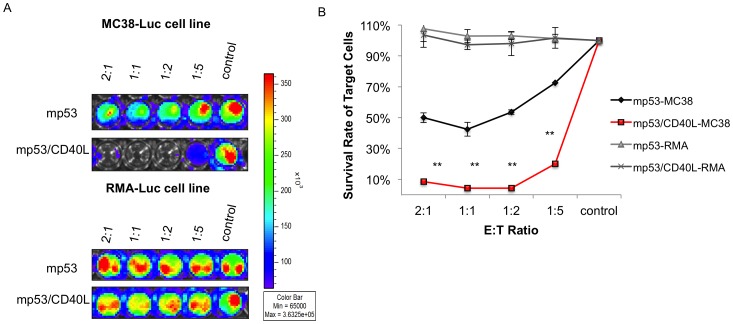
Characterization of the cytotoxic effect of mp53-specific CD8^+^ T cells from mice vaccinated with mp53/CD40L or mp53 DNA alone. (A) 5×10^4^ MC38-GFP-Luc cells (top) and RMA-GFP-Luc cells (a non-p53 expressing cell) (bottom) were seeded on 96-well plates for 12 hours. T cells from mp53 and mp53/CD40L DNA vaccinated mice were stimulated in vitro with mp53 CTL epitope (peptide 232) for one week with IL-2. The stimulated T cells were added to the plate via sequential titration E/T ratio (2∶1, 1∶1, 1∶2, 1∶5). The luciferase signal was examined after 16 hours using the IVIS Luminescence Imaging System 200 Series. (B) Plot depicting the percentage of target cell survival. Data presented as mean ± S.E. (**p<0.01).

In order to show that CD8^+^ T cells generated by vaccinated mice were antigen-specific, we performed an *in vitro* T cell killing assay using a luciferase-based bioluminescence imaging system. Splenocytes from mice vaccinated with DNA encoding mp53 or mp53/CD40L, were isolated and then stimulated *in vitro* with mp53 CTL epitope (aa 232–240) and IL-2 (2 IU/mL) for one week. The T cells were added to wells containing pre-seeded MC3-GFP-8Luc cells or RMA-GFP-Luc cells and were titrated to target cell ratio (E/T ratio). As expected, T cells from mp53 and mp53/CD40L vaccinated mice generated only limited clearance of RMA-GFP-Luc cells, which do not express p53 (**Fig. 4A and B**). Importantly, we also observed that mp53-specific T cells from mp53/CD40L vaccinated mice were capable of lysing MC38-GFP-Luc cells more effectively at lower E/T ratios (1∶1 and 1∶2) compared to those T cells from mice vaccinated with mp53 DNA only. These results indicate that mice vaccinated with DNA encoding mp53/CD40L were able to generate mp53-specific CD8^+^ T cells capable of killing mp53-expressing tumor cells.

### mp53-specific T cells expressed higher anti-apoptotic signals and lower pro-apoptotic signals following repeated CD40L stimulation from mp53-iTC-1/CD40L

**Figure 5:**
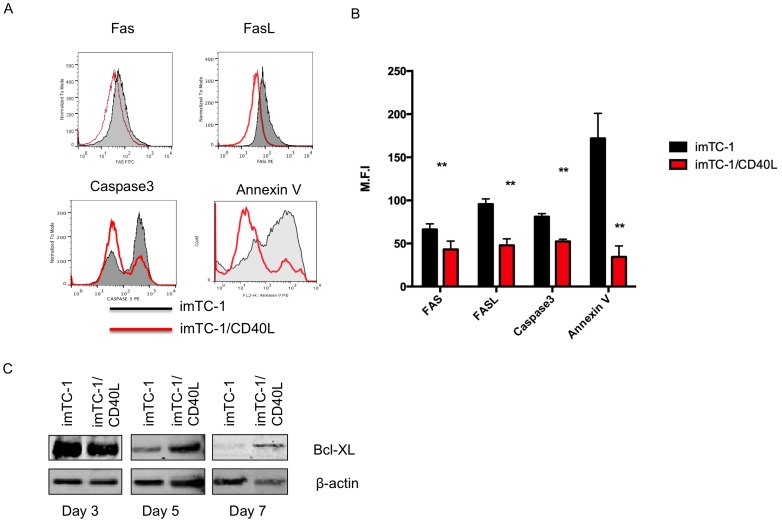
Characterization of anti- and pro-apoptotic signals in mp53-specific CD8+ T cells following mp53-iTC-1 or mp53-iTC-1/CD40L stimulation. (A) Flow cytometry analysis for Fas, FasL, caspase-3 and Annexin V. T cells from mice vaccinated with mp53 DNA followed by stimulation with mp53-iTC-1 or mp53-iTC-1/CD40L. (B) Bar graph depicting the MFI of the pro-apoptotic molecule on T cells (** p<0.01). (C) Bcl-XL protein expression by mp53-specific T cells was examined by western blot on days 3, 5 and 7 after stimulation with mp53-iTC-1 or mp53-iTC-1/CD40L.

We compared the Fas/FasL expression on T cells from mp53 DNA vaccinated mice after 7 days of stimulation with mp53-iTC-1 or mp53-iTC-1/CD40L cells. We observed a significant decrease in the Fas and FasL expression on T cells stimulated by mp53-iTC-1/CD40L (**Fig. 5A and B**) [36], [41]. We further compared the expression levels of pro-apoptotic signals, caspase 3 and Annexin V, in T cells stimulated by mp53-iTC-1 and mp53-iTC-1/CD40L. The T cells stimulated by mp53-iTC-1/CD40L demonstrated significantly lower median fluorescence intensity (MFI) of caspase 3 and Annexin V compared to T cells stimulated by mp53-iTC-1 (**Fig. 5A and B**). This phenomenon suggests that T cells may have enhanced survival after repeat stimulation by mp53-iTC-1/CD40L. Bcl-XL expression has been shown to be capable of inhibiting Fas-mediated apoptosis [41], [42]. Therefore, we collected T cell lysate on days 3, 5, and 7 after stimulation to evaluate Bcl-XL expression level by Western blot. As shown in **Fig. 5C**, on day 3, no significant difference in Bcl-XL expression was observed between mp53-iTC-1 and mp53-iTC-1/CD40L stimulated T cells. However, by day 5, the Bcl-XL levels on T cells stimulated by mp53-iTC-1 began to decrease compared to T cells stimulated by mp53-iTC-1/CD40L. These results suggest that repeat stimulation by tumor cells expressing CD40L increased survival signaling and decreased pro-apoptotic signaling in T cells. These results may account for why co-stimulation with CD40L rescues T cells from programmed cell death.

### mp53-specific CD8+ T cells had enhanced functional avidity and proliferation after co-stimulation with CD40L

**Figure 6:**
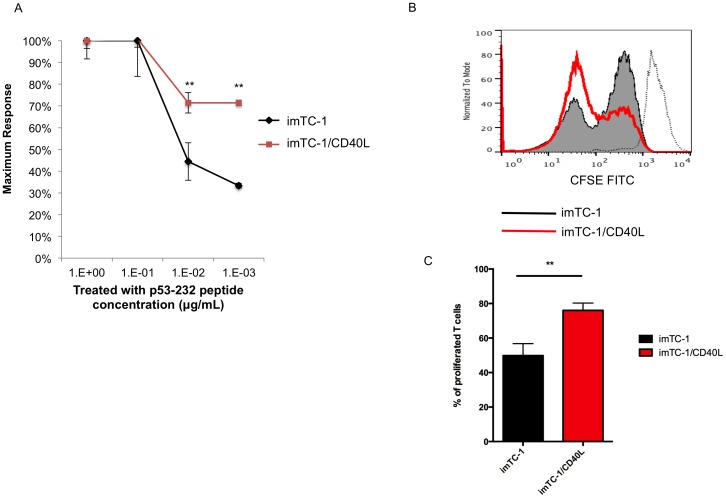
Characterization of T cell functional avidity and proliferation in the mp53 system. To evaluate T cell function, two groups of T cells were stimulated by titrated irradiated splenocytes loaded with mp53–232 peptide (1∼10^−3^ μg/mL). (**A**) The relative frequency of IFN-γ^+^ CD8^+^ T cells as a function of peptide concentration is depicted. (**B**) T cell proliferation assay. mp53-iTC-1 and mp53-iTC-1/CD40L stimulated mp53-specific T cells were stained with CFSE on day 1 prior to stimulation and then collected and analyzed by flow cytometry on day 3. A diluted CFSE signal indicates proliferation. (**C**) Bar graph depicting the percentage of proliferating T cells. Data presented as mean ± S.E. (**p<0.01).

Next we examined the difference in biological function between mp53-specific CD8+ T cells stimulated with mp53-iTC-1 or mp53-iTC-1/CD40L. We first assessed the functional avidity of mp53-iTC-1 and mp53-iTC-1/CD40L stimulated mp53-specific T cells by incubating them with p53 CTL epitope (aa 232–240) peptide in sequential titrations (1, 10^−1^, 10^−2^, 10^−3^ μg/mL). **Fig. 6A** shows that mp53-iTC-1/CD40L stimulated mp53-specific T cells generated a significantly higher percentage of CD8^+^ INF-γ^+^ cells compared to mp53-iTC-1 stimulated mp53-specific T cells (*p*<0.01), indicating increased functional avidity. Without peptide stimulation, no difference in T cell activation was observed between the groups. Next, we performed a T cell proliferation assay. T cells were stained with CFSE on day 1, prior to stimulation and then collected and analyzed by FACScan on day 3 [43]. **Fig. 6B and C** demonstrate that mp53-iTC-1/CD40L stimulated mp53-specific T cells had a lower CFSE signal than mp53-iTC-1 stimulated mp53-specific T cells, indicating increased proliferation. Taken together, these data indicate that mp53-iTC-1/CD40L stimulated mp53-specific T cells had enhanced functional avidity and proliferation compared to imTC-1 stimulated mp53-specific T cells.

### Adoptive transfer of splenocytes from Pmel TCR mice into irradiated host and vaccination with mGP100 DNA and CD40L DNA generates a significantly higher number of IFN-γ-producing antigen-specific T cells

**Figure 7:**
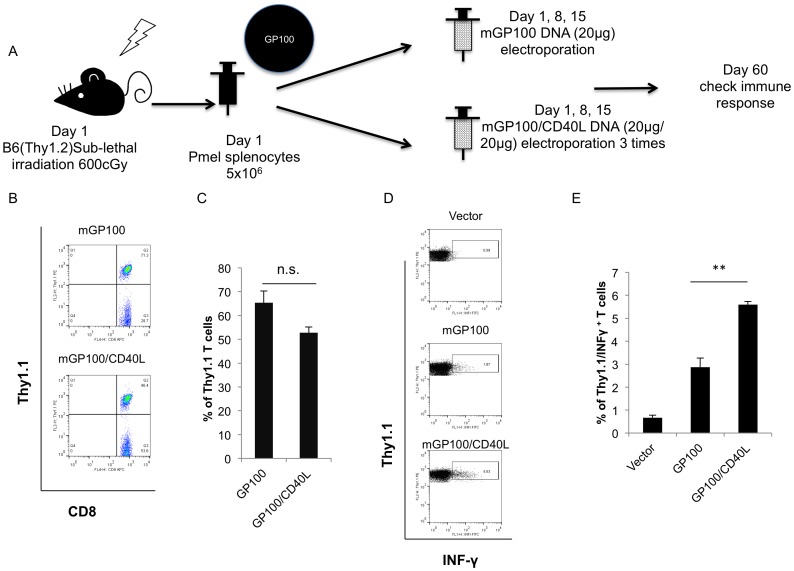
Characterization of mGP100-specific CD8^+^ T cell response following adoptive transfer of splenocytes from Pmel TCR mice into irradiated host and vaccination with mGP100 DNA with or without coadministration of CD40L DNA. (A) Schematic diagram depicting the adoptive transfer and vaccination schedule. After C57BL/6 mice (Thy1.2 background) were treated with 600cGy sub-lethal irradiation, 5×10^6^ splenocytes from Pmel TCR transgenic mice under Thy1.1 background were adoptively transferred into mice on day 1. The chimeric mice were vaccinated with mGP100 DNA (10 μg/mice) with or without coadministration of CD40L DNA (10 μg/mice) followed by electroporation on days 1, 8 and 15. 60 days after the adoptive transfer, splenocytes from vaccinated mice were isolated and incubated with mGP100_25–33_ peptide overnight. (B) Representative flow cytometry analysis. The presence of mGP100-specific CD8^+^ T cells was determined by gating the Thy1.1 population. (C) Bar graph depicting flow cytometry results. (D) Activated mGP100 T cells were determined by CD8 and intracellular IFN-γ staining followed by flow cytometry analysis. Representative flow cytometry analysis. (E) Bar graph depicting the percentage of IFN-γ/CD8^+^ cells. Data presented as mean ± S.E. (**p<0.01).

We further examined the ability of CD40-CD40L to improve antigen-specific CTL-mediated antitumor immune responses using the GP100 melanoma antigen system. In order to further understand the biology of mGP100 T cells, we amplified the T cell population by adoptively transferring 5×10^6^ splenocytes from Pmel-1 TCR mice (Thy1.1 background) into bone marrow-depleted C57BL/6 mice (Thy1.2 background) as previously described [38]. We examined the CD8^+^ T cells by vaccinating chimeric mice with pcDNA3-mGP100 or pcDNA3-mGP100/pcDNA3-CD40L DNA plasmids three times using the same dose and regimen as that described for the mp53 DNA vaccination strategy. The vaccination schedule is shown in **Fig. 7A**. 45 days after vaccination, mice generated around 50% mGP100-specific T cells; the proportion of Thy1.1 CD8+ T cells were not significantly different between groups of mice, as shown in **Fig. 7B and C**. To confirm the consistency of this system, we found that a significantly higher number of mGP100-specific CD8^+^ T cells from the chimeric mice vaccinated with mGP100/CD40L (mGP100/CD40L T cells) produced IFN-γ compared to those vaccinated with mGP100 T cells (p<0.01) **Fig. 7D and E**. This revealed that co-administration of CD40L in GP100 antigen setting generated more activated antigen-specific CD8^+^ T cells.

### In vitro stimulation with mGP100-iTC-1/CD40L generated a significantly higher number of IFN-γ expressing mGP100-specific CD8^+^ T cells compared to stimulation with mGP100-iTC-1

**Figure 8:**
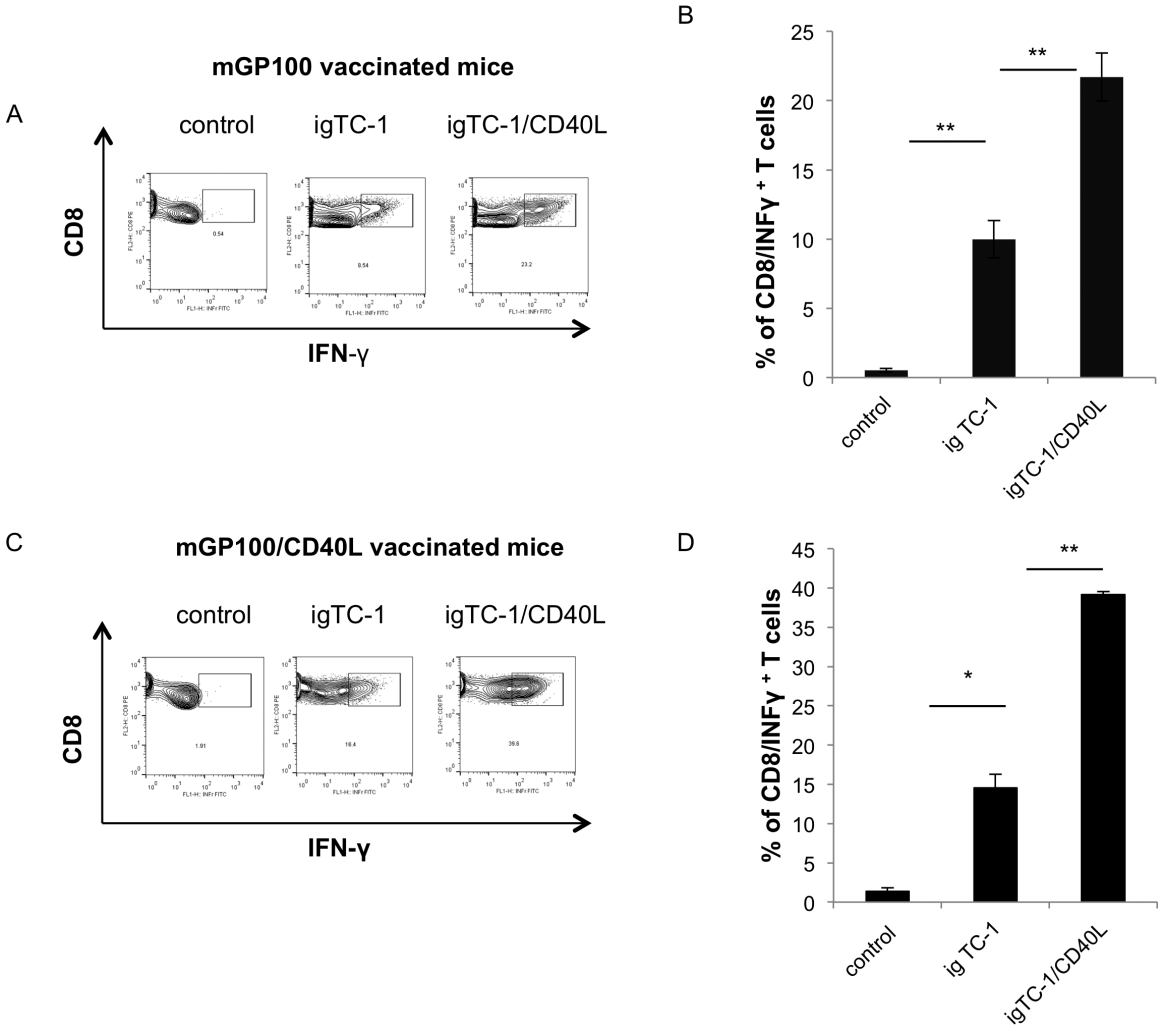
Characterization of mGP100-specific CD8^+^ T cell activation from mice vaccinated with mGP100/CD40L DNA or mGP100 DNA alone in vitro. (A) mGP100-specific T cells (generated from the chimeric mice vaccinated with mGP100 DNA), were incubated with mGP100-iTC-1 or mGP100-iTC-1/CD40L cells. mGP100 T cells were stained for CD8 and IFN-γ and analyzed by flow cytometry. Representative flow cytometry is depicted. (B) Bar graph depicting the percentage of IFN-γ^+^ mGP100 T cells. (C) mGP100-specific T cells from mGP100/CD40L DNA vaccinated chimeric mice were treated and analyzed. Representative flow cytometry is shown. (D) Bar graph depicting the percentage of IFN-γ^+^ mGP100/CD40L T cells. Data presented as mean ± S.E. (*p<0.05; **p<0.01).

We further stimulated the mGP100 T cells from mice vaccinated with pcDNA3-mGP100 with or without coadministration of pcDNA3-CD40L DNA *in vitro*. As shown in **Fig. 8A and B**, a significantly greater number of mGP100-specific T cells from the mice vaccinated with mGP100 DNA (mGP100 T cells) expressed IFN-γ when stimulated by mGP100-iTC-1/CD40L compared to those stimulated by mGP100-iTC-1 (*p*<0.01). Furthermore, as shown in **Fig. 8C and D**, we found that among the mGP100-specific CD8^+^ T cells from the mice vaccinated with mGP100/CD40L (mGP100/CD40L T cells) there were a significantly greater number producing IFN-γ when stimulated with mGP100-iTC-1/CD40L compared to mGP100-iTC-1 (*p*<0.01). These data indicate that costimulation with CD40L can enhance T cell functional avidity in different antigenic systems.

### CD8^+^ T cells from mGP100/CD40L vaccinated mice demonstrated enhanced mGP100-specific killing compared to those vaccinated with mGP100 alone

**Figure 9:**
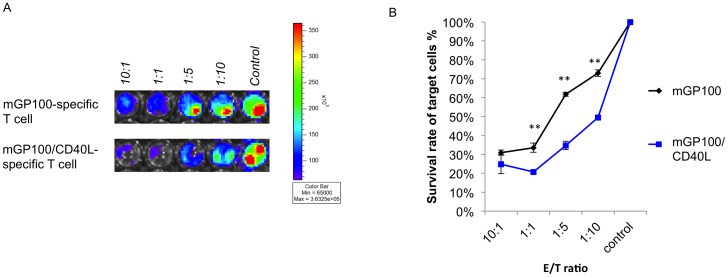
CD8^+^ T cells from mGP100/CD40L vaccinated mice demonstrated enhanced mGP100-specific killing compared to those vaccinated with mGP100 alone. (A) 1×10^5^ B16-Luc cells were seeded on 96-well plates for 12 hours. mGP100-specific T cells generated from mice vaccinated with mGP100 or mGP100/CD40L DNA plasmid were added to the plate via sequential titration E/T ratio (10, 1, 0.2, 0.1, control). Luciferase activity was examined after 16 hours. (B) Bar graph depicting the survival rate of target cells. Data presented as mean ± S.E. (**p<0.01).

In order to test whether co-administration with CD40L DNA could enhance antigen-specific CD8^+^ T cell mediated killing in the GP100 antigenic system, we performed an *in vitro* CD8^+^ T cell-mediated killing assay using a luciferase-based bioluminescence imaging system. mGP100-specific CD8^+^ T cells were generated from mGP100 DNA or mGP100/CD40L DNA vaccinated chimeric mice were added to pre-seeded B16-Luc cells and titrated to different effector to target cell ratios (E/T ratio). The mGP100-specific CD8^+^ T cells from mice vaccinated with mGP100/CD40L DNA demonstrated significantly enhanced killing compared to mGP100-specific CD8^+^ T cells from mice vaccinated with mGP100 DNA only (**Fig. 9A and B**). These results indicate that co-administration of CD40L DNA with mGP100 antigen-specific DNA vaccines can increase mGP100-specific CD8^+^ T cell-mediated killing in the mGP100 system.

### mGP100-specific T cells had improved functional avidity and proliferation after co-stimulation with CD40L

**Figure 10:**
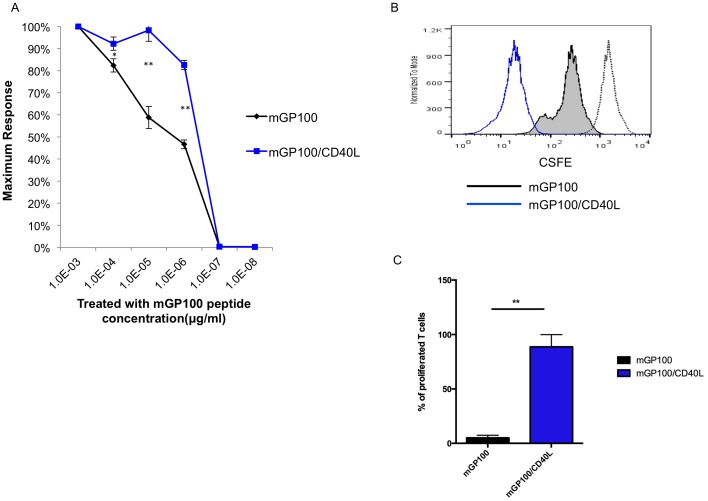
Characterization of T cell functional avidity and proliferation in the mGP100 system. (**A**) mGP100-specific T cells from mice vaccinated with mGP100 or mGP100/CD40L DNA plasmids were stimulated by titrations of mGP100 peptide (10^−3^, 10^−4^, 10^−5^, 10^−6^,10^−7^ and 10^−8^ μg/mL), and then stained for CD8 and IFN-γ and analyzed by flow cytometry. The relative frequency of IFN-γ^+^ CD8^+^ T cells as a function of peptide concentration is depicted. (**B**) T cell proliferation assay. mGP100-specific T cells from mice vaccinated with mGP100 or mGP100/CD40L DNA plasmids were stained with CFSE on day 1, prior to stimulation and then collected and analyzed by flow cytometry on day 3 after incubation with mGP100-iTC-1 cells. A diluted CFSE signal indicates proliferation. (**C**) Bar graph depicting the proliferated T cells. Data presented as mean ± S.E. (*p<0.05; **p<0.01).

mGP100-specific T cells from mice vaccinated with mGP100 or mGP100/CD40L DNA plasmid were stimulated by titrations of mGP100 peptide (10^−3^, 10^−4^, 10^−5^, 10^−6^,10^−7^ and 10^−8^ μg/mL). The IFN-γ^+^ mGP100-specific CD8^+^ T cells were normalized to a concentration 10^−3^ μg/mL of mGP100 peptide in both groups. **Fig. 10A** shows that 82.5% of the mGP100-specific T cells from mice vaccinated with mGP100/CD40L DNA plasmids still produced IFN-γ^+^ when the peptide concentration was titrated to 10^−6^ μg/mL while the activation of mGP100-iTC-1 stimulated mGP100-specific T cells dropped to 46.2% (*p*<0.01). This phenomenon of increased functional avidity is consistent with what we observed in the mp53-specific CD8^+^ T cell system. As shown in **Fig. 10B and C**, the CFSE proliferation assay indicated that mGP100-specific CD8^+^ T cells from mice vaccinated with mGP100/CD40L DNA plasmid proliferated to a greater degree than mGP100-specific CD8^+^ T cells from mice vaccinated with mGP100 DNA plasmid. Our data indicate that mGP100-specific CD8^+^ T cells from mice vaccinated with mGP100/CD40L develop higher functional avidity and increased proliferative ability compared to mGP100-specific CD8^+^ T cells from mice vaccinated with mGP100 alone.

### Antigen-specific CD8^+^ T cells, following repeated CD40L co-stimulation, expressed higher anti-apoptotic signaling, and lower pro-apoptotic signaling

**Figure 11:**
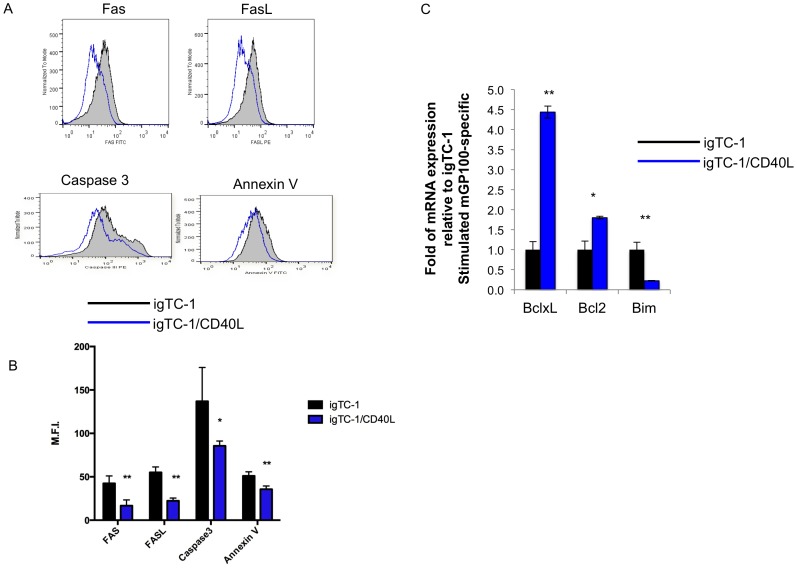
Characterization of anti- and pro-apoptotic signals expression on mGP100-iTC-1 and mGP100-iTC-1/CD40L stimulated mGP100-specific CD8^+^ T cells. (A) After repeated stimulation, mGP100-iTC-1 and mGP100-iTC-1/CD40L stimulated mGP100-specific T cells were analyzed for Fas and FasL cell surface expression by flow cytometry. mGP100-iTC-1 and mGP100-iTC-1/CD40L stimulated mGP100-specific T cells were stained for pro-apoptotic signals, caspase-3 and Annexin V, and analyzed by flow cytometry. (B) Bar graph depicting the MFI of pro-apoptotic signal molecule on T cells. (C) mRNA levels of Bcl-XL, Bcl-2 and Bim from both mGP100-iTC-1 and mGP100-iTC-1/CD40L stimulated mGP100-specific T cells were analyzed by quantitative real-time PCR. Bar graph shows mRNA expression standardized to mGP100-iTC-1 stimulated mGP100-specific T cells. Data presented as mean ± S.E. (*p<0.05; **p<0.01).

To test whether repeated CD40L stimulation would change the T cell phenotype in the mGP100 system, we generated an mGP100-specific T cell line as mentioned above and stimulated cells *in vitro* with mGP100-iTC-1 or mGP100-iTC-1/CD40L for 5 cycles. We stained for the pro-apoptotic signals Fas, FasL, caspase 3, and Annexin V in both groups of T cells. We observed that mGP100-iTC-1/CD40L stimulated mGP100-specific T cells expressed fewer pro-apoptotic signals compared to mGP100-iTC-1 stimulated mGP100-specific T cells (**Fig. 11A and B**). We also examined the mRNA levels of Bcl-XL, Bcl-2 (anti-apoptotic signals) and Bim (pro-apoptotic signal) in both T cell populations (**Fig. 11C**). mGP100-iTC-1/CD40L stimulated mGP100-specific had higher levels of expression of both Bcl-XL and Bcl-2 mRNA but had a lower expression level of Bim mRNA compared to mGP100-iTC-1 stimulated mGP100-specific T cells. These data imply that this T cell population has the potential to proliferate and survive longer under co-stimulation with CD40L.

### mGP100-iTC-1/CD40L stimulated mGP100-specific T cells expressed a higher density of TCR Vβ13 compared to mGP100-iTC-1 stimulated mGP100-specific T cells

**Figure 12:**
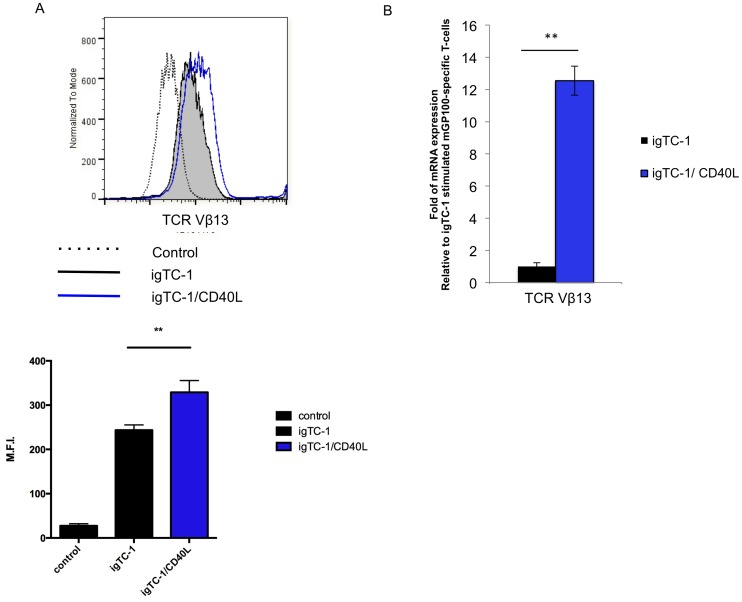
Expression of TCR Vβ13 on T cells following repeated stimulation with CD40L. (**A**) mGP100-iTC-1 and mGP100-iTC-1/CD40L stimulated mGP100-specific T cells were stained for TCR Vβ13 on day 5 after stimulation and analyzed by flow cytometry. (**B**) Bar graph depicting the MFI of TCR Vβ13 expressing on T cells. (**C**) Bar graph showing the expression level of TCR Vβ13 mRNA relative to mGP100-iTC-1 stimulated mGP100-specific T cells. Data presented as mean ± S.E. (**p<0.01).

A unique characteristic of mGP100-specific CD8^+^ T cells generated from Pmel-1 TCR transgenic mice is that they have a single clone of TCR Vβ13 [20]. This allowed us to clearly trace the TCR Vβ13 expression on CD8^+^ T cells. We collected both groups of T cells on day 5, post-stimulation. **Fig. 12A-B** shows that mGP100-iTC-1/CD40L stimulated mGP100-specific T cells had a higher density of TCR Vβ13 expression compared to mGP100-iTC-1 stimulated mGP100-specific T cells. We further examined the mRNA levels of both groups of T cells. The TCR Vβ13 mRNA expression levels were consistent with flow cytometric data, showing significantly greater TCR Vβ13 mRNA in mGP100-iTC-1/CD40L stimulated mGP100-specific T cells compared to mGP100-iTC-1 stimulated mGP100-specific T cells (*p*<0.01) (**Fig. 12C**). These data suggest that co-stimulation with CD40L results in higher expression of T cell receptors on CD8^+^ T cells.

## Discussion

In the current study, we successfully utilized a DNA vaccination strategy combining an antigen-specific DNA and DNA encoding CD40L to improve antigen-specific CD8^+^ T cell immune responses against endogenous tumor antigens. We found that following repeated CD40L co-stimulation, low avidity CD8^+^ T cells exhibited improved functional avidity, tumor killing, and proliferation. These CD8^+^ T cells demonstrated higher TCR expression on their surface. In addition, high avidity CD8^+^ T cells expressed increased anti-apoptotic signals and decreased pro-apoptotic signals. Furthermore, we found that after repeated CD40L stimulation, memory T cells demonstrated higher stem cell-like phenotypes. In this way, the vaccine generated a robust immune response breaking immunological tolerance, thereby controlling p53-expressing tumors. Similar results were observed in the GP100 antigenic system. Therefore, administration of DNA encoding a TAA and CD40L DNA via intramuscular injection followed by electroporation can break immune tolerance. Overall, our approach may have wide ranging applications in treating diseases affected by similar tolerance issues.

The current study incorporated two models, p53 and GP100. p53, as stated above, is a TAA that is upregulated in more than fifty percent of human cancers. Therefore, the p53 model was utilized as verification of the TAA/CD40L DNA vaccine's translational value. Furthermore, the employment of the melanoma antigen GP100, a well-studied TAA, allows a clearer dissection of the mechanism involved. We used pmel-1 TCR transgenic mice that carry a rearranged T cell receptor transgene specific for the mouse homologue of GP100. Therefore, the majority of CD8^+^ T cells in the transgenic mice express a transgenic TCR. This allowed the determination that CD8^+^ T cells were chiefly responsible for the tumor clearance observed within the p53 model. As verification, depletion experiments were performed in which anti-CD4, anti-CD8, or anti-NK1.1 Antibodies were consistently administered prior to and during MC38 tumor challenge. The results showed that mice injected with anti-CD8 antibodies performed significantly worse in comparison to the anti-CD4 and anti-NK1.1 treated mice.

When targeting TAAs in cancer immunotherapeutics, overcoming immune tolerance is a major challenge. One approach to do so is to increase the avidity of antigen-specific CD8^+^ T cells to improve their killing efficacy. It has been shown that electroporation of mature DCs with RNA encoding CD40L, promotes DCs to prime naïve CD28^+^ CTLs in vitro thereby inducing high-avidity TCR expression, IL-2 secretion, and an effector memory phenotype [44]. This suggests that targeting CD40 may serve as an ideal mechanism to generate such high-avidity antigen-specific T cells, and some studies have done just that. For example, Cho and Celis have demonstrated that a vaccination approach using synthetic peptides representing CTL epitopes, TLR agonists and anti-CD40 antibodies was able to generate large quantities of high-avidity antigen-reactive T cells capable of killing tumor cells [45]. Additionally, Aranda et al were able to overcome immune tolerance in the B16-OVA tumor model using the TLR7 and TLR3 ligands, anti-CD40 agonistic antibodies and antigen targeted to DCs [46]. These previous studies as well as the current approach support the further clinical translation of cancer therapies targeting CD40.

An important characteristic to achieve with tumor immunotherapy is the generation of long-lasting immunity. Such immunological memory is now understood to be mediated, at least in part, by a stem cell-like population of memory T lymphocytes (for review see [47]. Gattinoni et al characterized this subset of T cells when they found that Wnt signaling promoted the generation of self-renewing multipotent CD8^+^ memory T cells [48], [49]. In the current study, we found that our strategy, directly activating T cells with CD40L, enhanced the expression of anti-apoptotic signals by antigen-specific CD8^+^ T cells (**Figs. 5** and **11**). Our observation corresponds with the previous finding that CD40 signal transduction and subsequent ERK1/2 activation is required for apoptosis rescue in monocytes (for review see [50]). The expression of anti-apoptotic proteins is an important feature of memory T cells. This suggests that the current strategy may be ideal for the generation of long-lasting immunity.

One of the mechanisms by which the current approach increases the avidity of antigen-specific CD8^+^ T cells may be that it induces high TCR expression. Previously, it has been shown that CD8^+^ T cell reactivity to weak ligands is in large part determined by TCR density [51]. In the current study, we found that mGP100-iTC-1/CD40L stimulated mGP100-specific T cells had a higher density of TCR Vβ13 mRNA and cell surface protein expression compared to mGP100-iTC-1 stimulated mGP100-specific T cells (**Fig. 12**). It is possible that the lower level of TCR Vβ13 expression on T cells cultured in absence of the CD40L was the result of their pro-apoptotic phenotype. We also observed this phenomenon in the current study; mp53-iTC-1/CD40L stimulated mp53-specific T cells had enhanced proliferation (**Fig. 6**). This was also the case in the mGP100 system (**Fig. 10**).

In summary, CD40L serves as an important costimulatory molecule responsible for regulating the pathways that provide signaling to T cells. Our study indicates that CD40L is critical in generating effective antigen-specific immune responses in a weakly immunogenic TAA setting. Persistent stimulation with CD40L has the potential to enhance the functional avidity and proliferation of T cells to perform better functionally, breaking immunological tolerance and thereby achieving tumor control. Thus, the employment of a DNA construct encoding CD40L for the development of DNA vaccines represents a potentially promising approach for breaking immune tolerance when endogenous tumor antigens are targeted. The underlying mechanism requires further elucidation in order to improve the translational value.

## Supporting Information

Figure S1 **Expression of CD40L on APCs transfected with CD40L.** TC-1 cells were used as APCs. TC-1 cells were transfected with or without DNA plasmid encoding GFP-CD40L. TC-1 and TC-1-GFP-CD40L cells were then stained with anti-CD40L antibody and analyzed by flow cytometry.
(TIF)

Figure S2 **Characterization of the population of mp53-specific CD8^+^ T cells.** After splenocytes from mp53 DNA vaccinated mice were stimulated with imTC-1 or imTC-1/CD40L, cells were stained with anti-CD8 antibody and H-2K^b^ KYMCNSSCM pentamer for sorting. Representative flow cytometry analyses are shown.
(TIF)

Figure S3 **Antigen-specific T cell response following CD40L driven co-stimulation in vivo and in vitro.** C57BL/6 mice were vaccinated twice with DNA plasmid, either pcDNA3-CRTE7 or pcDNA3-CRTE7/CD40L, by gene gun once every other week. Peripheral blood from the tail veins of mice was obtained and stained by E7-MHC class I tetramer and anti-CD8 Ab. (**A**) Representative flow cytometry. (**B**) Bar graph depicting the percentage of E7 tetramer/CD8^+^ T cells among splenocytes. (**C**) Splenocytes from treated mice were stimulated with E7 specific peptide overnight and stained by anti-CD8 Ab and anti-IFN-γ. Representative flow cytometry analysis. (**D**) Bar graph showing the number of CD8/IFN-γ^+^ cells among splenocytes. (**E**) In vitro, E7-specific T cells were incubated with TC-1 or TC-1/CD40L. Representative flow cytometry analysis (**F**) Bar graph showing the percentage of E7-specific CD8^+^ T cells. Data presented as mean ± S.E.
(TIF)

Figure S4 **Tumor volume of mice in prevention model.** (**A**) Mice (n = 5) were immunized with various DNA vaccines (mp53, CD40L, or mp53/CD40L) three times at one week intervals and then challenged with MC38 (2×10^5^/mouse). 1 week later, mice were monitored for survival following tumor challenge. Tumor volume was measured weekly with digital calipers (**B**) Mice (n = 5) were immunized with mp53/CD40L DNA vaccine via intramuscular injection with electroporation using the same regimens and challenged with 2×10^5^ MC38 cells per mouse. Anti-CD4, anti-CD8, anti-NK1.1 antibodies (100 μg/mouse) were administered every other day, beginning one week before tumor challenge. Following tumor challenge, antibodies were administered every 7 days and the treatment was terminated 30 days after tumor challenge. In vivo antibody depletion experiments in mice vaccinated with mp53/CD40L DNA plasmid. Tumor volume was measured weekly with digital calipers. Data are expressed as volume ± S.E. (**p<0.01).
(TIF)

Figure S5 **Tumor volume of mice in therapeutic model.** (**A**) Schematic diagram depicts tumor challenge and the vaccination schedule. Mice (n = 5) were challenged with MC38 (2×10^5^/mouse) and then immunized with various DNA vaccines (vector, mp53, or mp53/CD40L) on days 3, 8 and 11. (**B**) Tumor volume was measured weekly with digital calipers. Data are expressed as volume ± S.E. (**p<0.01). The line graph depicts the tumor volume in various treatment regimens.
(TIF)
