# Supplementary figures and images for: Direct T Cell Activation via CD40 Ligand Generates High Avidity CD8+ T Cells Capable of Breaking Immunological Tolerance for the Control of Tumors
Source: PLoS One. 2014 Mar 24;9(3):e93162. doi: 10.1371/journal.pone.0093162 (PMC3963987; doi:10.1371/journal.pone.0093162)

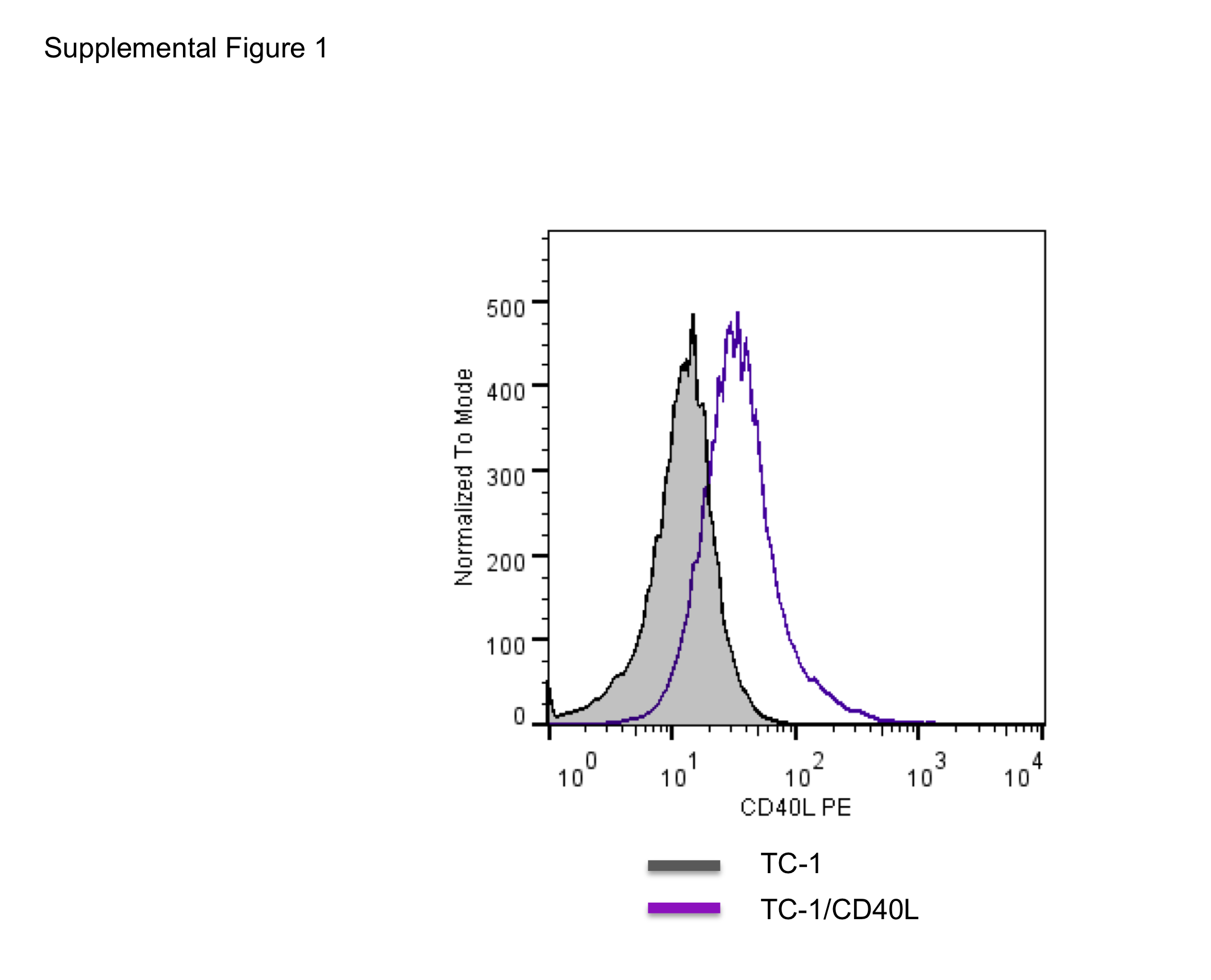

Supplement: Figure S1 — Expression of CD40L on APCs transfected with CD40L. TC-1 cells were used as APCs. TC-1 cells were transfected with or without DNA plasmid encoding GFP-CD40L. TC-1 and TC-1-GFP-CD40L cells were then stained with anti-CD40L antibody and analyzed by flow cytometry. (TIF) [file pone.0093162.s001.tif]

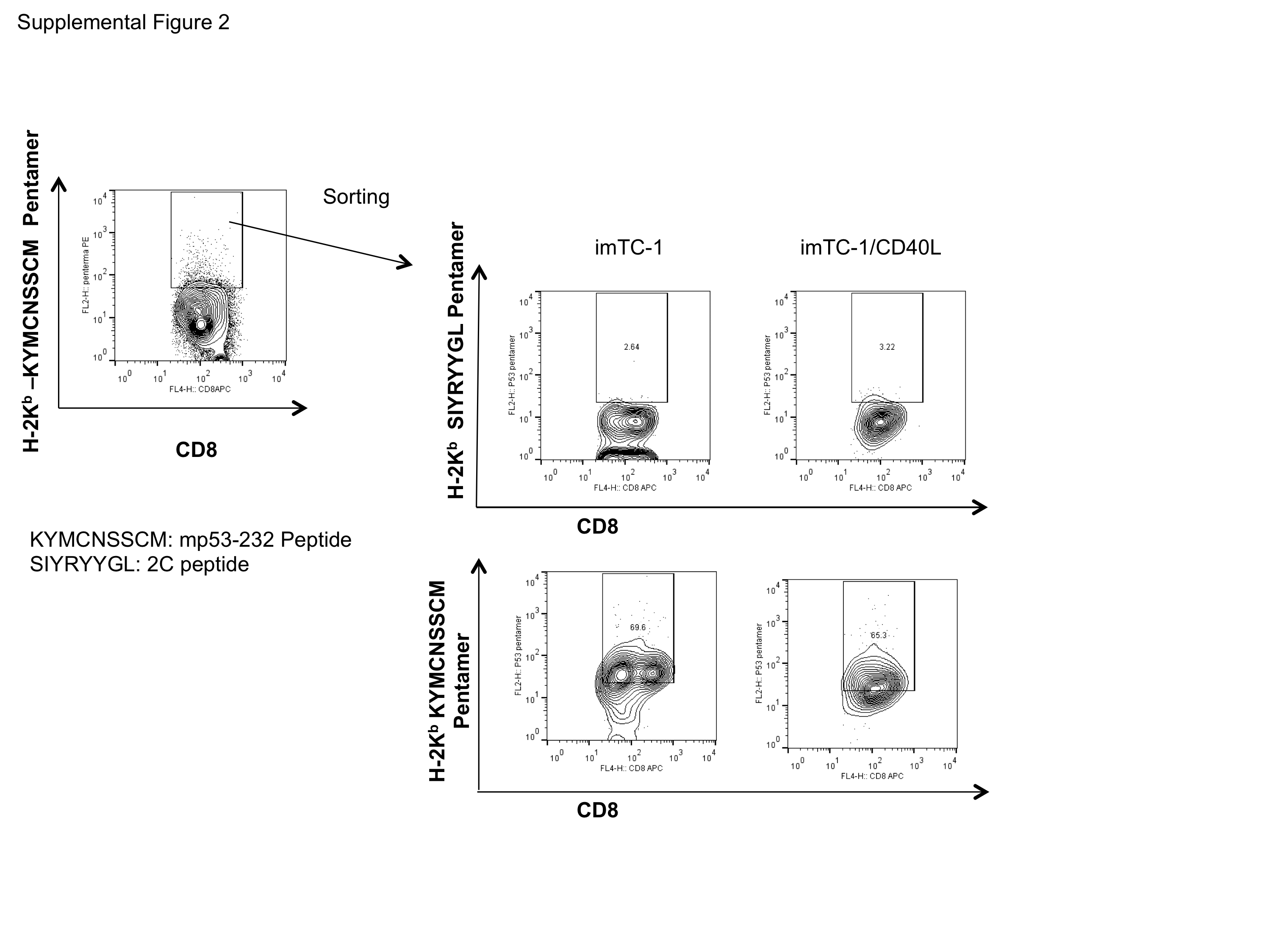

Supplement: Figure S2 — Characterization of the population of mp53-specific CD8+ T cells. After splenocytes from mp53 DNA vaccinated mice were stimulated with imTC-1 or imTC-1/CD40L, cells were stained with anti-CD8 antibody and H-2Kb KYMCNSSCM pentamer for sorting. Representative flow cytometry analyses are shown. (TIF) [file pone.0093162.s002.tif]

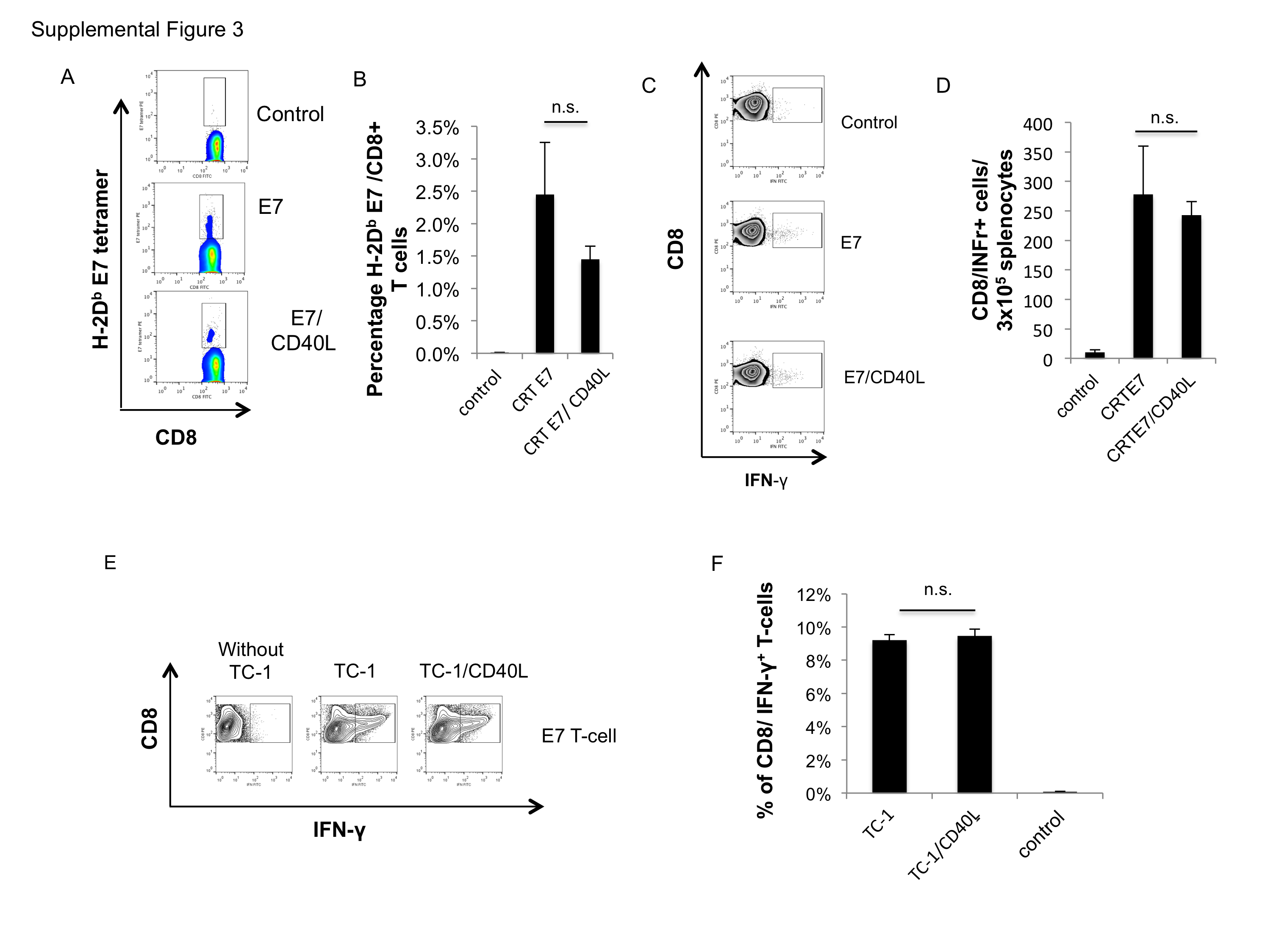

Supplement: Figure S3 — Antigen-specific T cell response following CD40L driven co-stimulation in vivo and in vitro. C57BL/6 mice were vaccinated twice with DNA plasmid, either pcDNA3-CRTE7 or pcDNA3-CRTE7/CD40L, by gene gun once every other week. Peripheral blood from the tail veins of mice was obtained and stained by E7-MHC class I tetramer and anti-CD8 Ab. (A) Representative flow cytometry. (B) Bar graph depicting the percentage of E7 tetramer/CD8+ T cells among splenocytes. (C) Splenocytes from treated mice were stimulated with E7 specific peptide overnight and stained by anti-CD8 Ab and anti-IFN-γ. Representative flow cytometry analysis. (D) Bar graph showing the number of CD8/IFN-γ+ cells among splenocytes. (E) In vitro, E7-specific T cells were incubated with TC-1 or TC-1/CD40L. Representative flow cytometry analysis (F) Bar graph showing the percentage of E7-specific CD8+ T cells. Data presented as mean ± S.E. (TIF) [file pone.0093162.s003.tif]

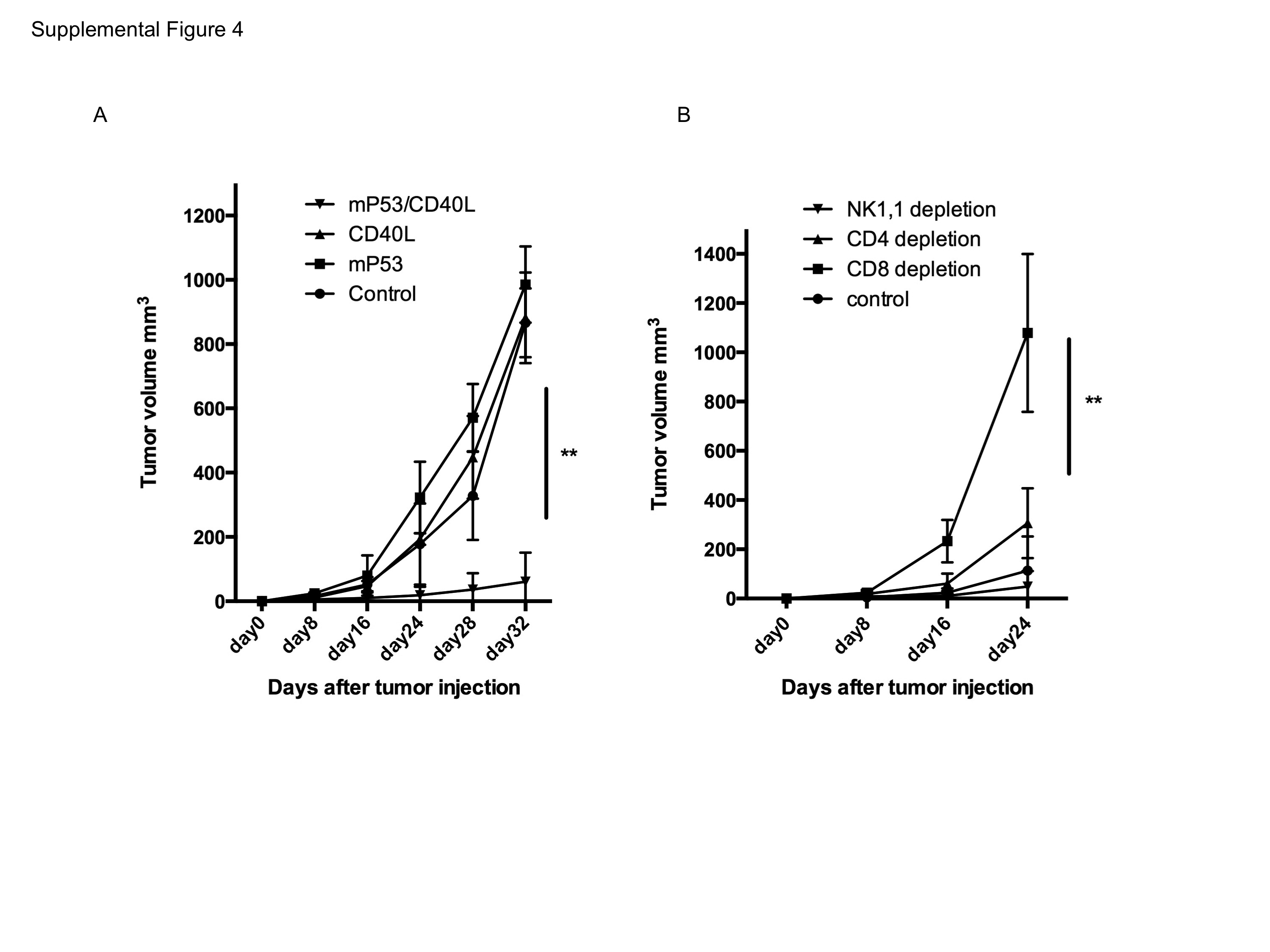

Supplement: Figure S4 — Tumor volume of mice in prevention model. (A) Mice (n = 5) were immunized with various DNA vaccines (mp53, CD40L, or mp53/CD40L) three times at one week intervals and then challenged with MC38 (2×10^5^/mouse). 1 week later, mice were monitored for survival following tumor challenge. Tumor volume was measured weekly with digital calipers (B) Mice (n = 5) were immunized with mp53/CD40L DNA vaccine via intramuscular injection with electroporation using the same regimens and challenged with 2×10^5^ MC38 cells per mouse. Anti-CD4, anti-CD8, anti-NK1.1 antibodies (100 μg/mouse) were administered every other day, beginning one week before tumor challenge. Following tumor challenge, antibodies were administered every 7 days and the treatment was terminated 30 days after tumor challenge. In vivo antibody depletion experiments in mice vaccinated with mp53/CD40L DNA plasmid. Tumor volume was measured weekly with digital calipers. Data are expressed as volume ± S.E. (**p<0.01). (TIF) [file pone.0093162.s004.tif]

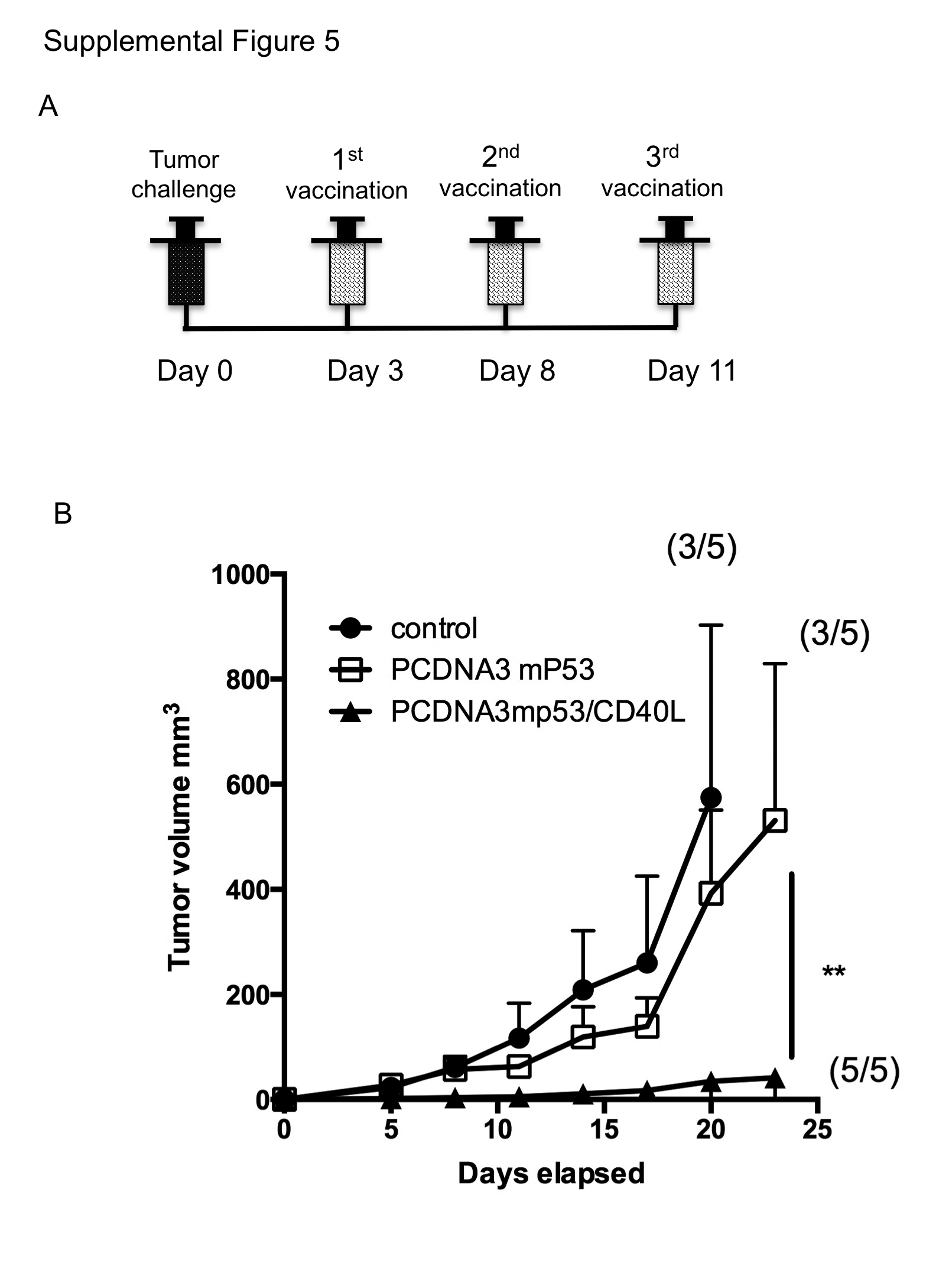

Supplement: Figure S5 — Tumor volume of mice in therapeutic model. (A) Schematic diagram depicts tumor challenge and the vaccination schedule. Mice (n = 5) were challenged with MC38 (2×10^5^/mouse) and then immunized with various DNA vaccines (vector, mp53, or mp53/CD40L) on days 3, 8 and 11. (B) Tumor volume was measured weekly with digital calipers. Data are expressed as volume ± S.E. (**p<0.01). The line graph depicts the tumor volume in various treatment regimens. (TIF) [file pone.0093162.s005.tif]
